# P-1496. Retrospective Study of Ceftazidime-Avibactam Treatment for Multidrug-Resistant Gram-Negative Bacterial Infections in King Chulalongkorn Memorial Hospital

**DOI:** 10.1093/ofid/ofae631.1666

**Published:** 2025-01-29

**Authors:** Kamonwan Jutivorakool, Rongpong Reinprayoon

**Affiliations:** King Chulalongkorn Memorial Hospital, Bangkok, Krung Thep, Thailand; Faculty of Medicine, Chulalongkorn University, Bangkok, Krung Thep, Thailand

## Abstract

**Background:**

Antimicrobial resistance, particularly among Enterobacterales, poses a significant healthcare threat. With the rise of carbapenem-resistant strains, alternative treatment strategies are necessary. This study explores the clinical experience of using ceftazidime-avibactam (CAZ-AVI) in treating infections caused by multidrug-resistant Gram-negative bacteria (MDR-GNB), including carbapenem-resistant pathogens.

**Table 1.**
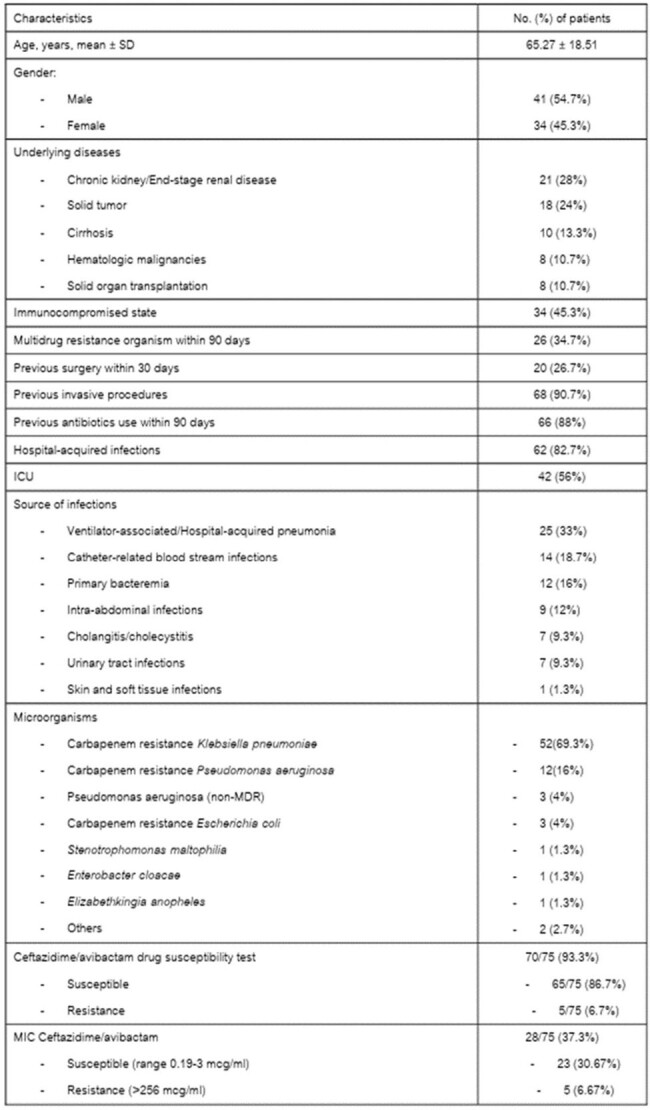
Baseline characteristics of the patients who received CAZ/AVI

**Methods:**

A single-center retrospective observational study was conducted at King Chulalongkorn Memorial Hospital, Thailand, from Jan. 2019 to Dec. 2022. The study included adult patients aged ≥18 years, who receiving ≥48 hours of CAZ-AVI for documented MDR-GNB infections. Data on demographics, comorbidities, treatment regimens, and outcomes were extracted from electronic medical records. The primary outcome was all-cause mortality at 14 days. Secondary objectives included clinical and microbiological responses at 14 days, and all-cause mortality at 28 days.

**Table 2.**
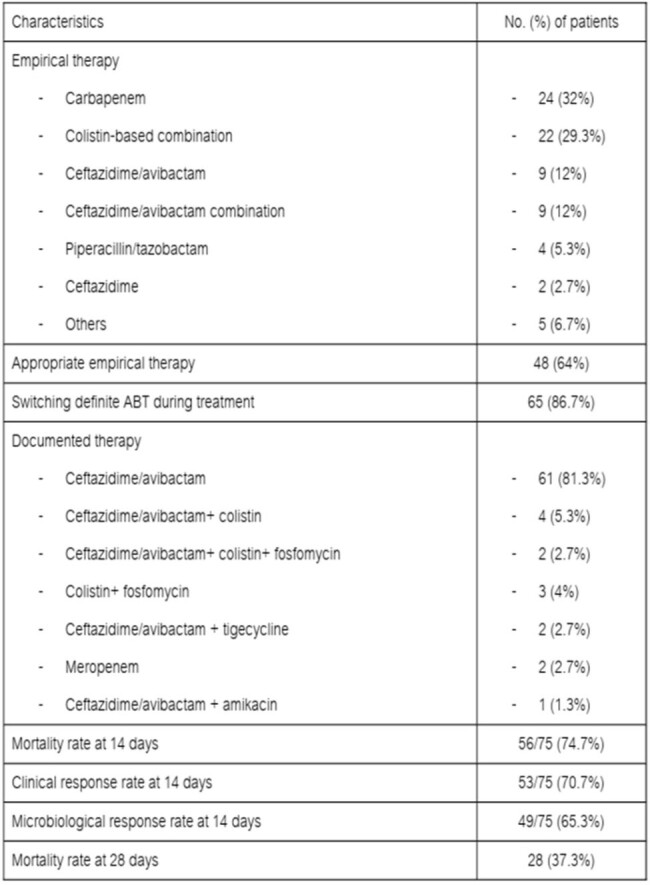
Clinical outcomes of the patients who received CAZ/AVI

**Results:**

Among 85 patients receiving CAZ-AVI, 75 met inclusion criteria. The mean age was 65.27 years, with 54.7% being male. Hospital-acquired infections accounted for 82.7%, and 56% were ICU patients. Underlying conditions included chronic kidney diseases/end-stage renal diseases (28%), solid tumors (24%), cirrhosis (13.3%), hematologic malignancies (10.7%), and solid organ transplantation (10.7%). *K. pneumoniae* was the most common organism (69.3%), followed by *P. aeruginosa* (20%). All (100%) of *K. pneumoniae* and *E. coli* were carbapenem resistance. CAZ-AVI susceptibility was observed in 93.6% of isolates. The 14-day all-cause mortality rate was 25.3%. Clinical and microbiological response rates were 70.7% and 65.3%, respectively. Infection by CAZ-AVI-susceptible organisms correlated with lower mortality rates (p=0.017). No adverse drug effects were reported.

**Table 3.**
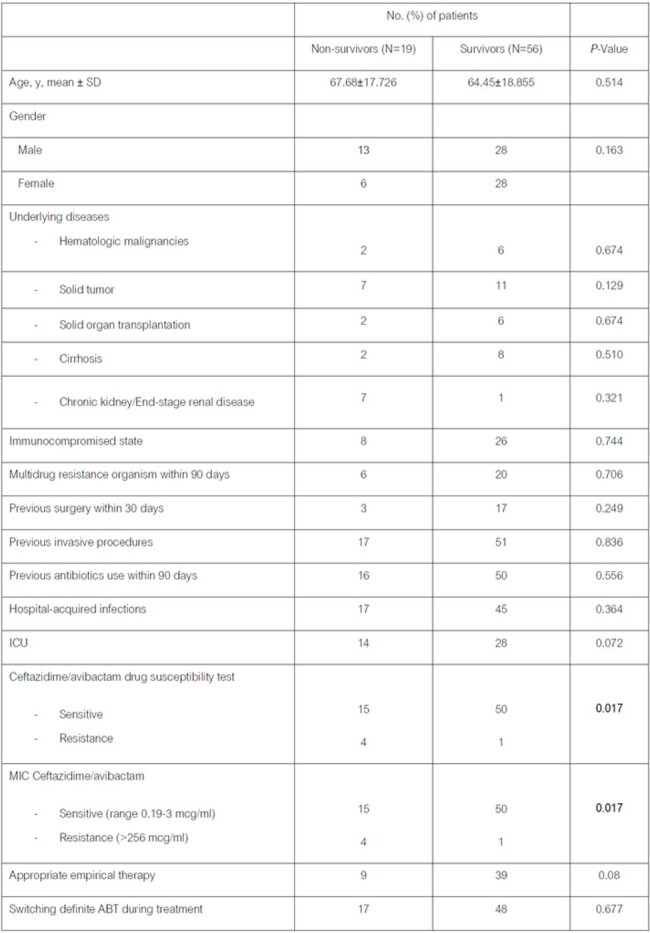
Clinical outcomes of the patients who received CAZ/AVI

**Conclusion:**

This study highlights the promising role of CAZ-AVI in treating MDR-GNB infections, offering an alternative to conventional therapies. While challenges remain, including the complexity of empirical therapy and variations in patient outcomes, CAZ-AVI demonstrates efficacy with a favorable safety profile.

**Disclosures:**

**KAMONWAN JUTIVORAKOOL, MD, Msc**, Pfizer, Thailand: Grant/Research Support|Pfizer, Thailand: Honoraria **Rongpong Reinprayoon, MD, MSc**, Pfizer: Grant/Research Support

